# Muscles in “Concert”: Study of Primary Motor Cortex Upper Limb Functional Topography

**DOI:** 10.1371/journal.pone.0003069

**Published:** 2008-08-27

**Authors:** Jean-Marc Melgari, Patrizio Pasqualetti, Flavia Pauri, Paolo Maria Rossini

**Affiliations:** 1 Neurology, University Campus Bio-Medico, Rome, Italy; 2 Medical Statistics & Information Technology, AFaR, Fatebenefratelli Hospital, Isola Tiberina, Rome; 3 Casa di Cura SAN RAFFAELE Cassino and IRCCS SAN RAFFAELE, Pisana, Rome, Italy; 4 Department of Neurology and ORL, Università La Sapienza, Rome, Italy; Columbia University, United States of America

## Abstract

**Background:**

Previous studies with Transcranial Magnetic Stimulation (TMS) have focused on the cortical representation of limited group of muscles. No attempts have been carried out so far to get simultaneous recordings from hand, forearm and arm with TMS in order to disentangle a ‘functional’ map providing information on the rules orchestrating muscle coupling and overlap. The aim of the present study is to disentangle functional associations between 12 upper limb muscles using two measures: cortical overlapping and cortical covariation of each pair of muscles. Interhemispheric differences and the influence of posture were evaluated as well.

**Methodology/Principal Findings:**

TMS mapping studies of 12 muscles belonging to hand, forearm and arm were performed. Findings demonstrate significant differences between the 66 pairs of muscles in terms of cortical overlapping: extremely high for hand-forearm muscles and very low for arm vs hand/forearm muscles. When right and left hemispheres were compared, overlapping between all possible pairs of muscles in the left hemisphere (62.5%) was significantly higher than in the right one (53.5% ).

The arm/hand posture influenced both measures of cortical association, the effect of Position being significant [p = .021] on overlapping, resulting in 59.5% with prone vs 53.2% with supine hand, but only for pairs of muscles belonging to hand and forearm, while no changes occurred in the overlapping of proximal muscles with those of more distal districts.

**Conclusions/Significance:**

Larger overlapping in the left hemisphere could be related to its lifetime higher training of all twelve muscles studied with respect to the right hemisphere, resulting in larger intra-cortical connectivity within primary motor cortex. Altogether, findings with prone hand might be ascribed to mechanisms facilitating coupling of muscles for object grasping and lifting -with more proximal involvement for joint stabilization- compared to supine hand facilitating actions like catching. TMS multiple-muscle mapping studies permit a better understanding of motor control and ‘plastic’ reorganization of motor system.

## Introduction

About 30 years ago a revolutionary technique was introduced which allowed stimulation of the human brain through the skull with non-invasive, high-intensity, extremely brief electric pulses [1]–[5], followed –few years later- by a new device that employs strong and brief, time-varying magnetic fields able to induce electric currents flowing within the brain without any discomfort [6]; for a review see [7]. Since then, focal types of stimulating coils able to activate relatively small cortical areas allowing cortical mapping were developed [8]. Therefore, focal TMS to different cells of a grid overlying the primary motor cortex (M1) while recording electromyographic (EMG) responses from the contralateral ‘target’ muscles, made it possible to obtain reliable maps of cortical motor output in awake and cooperative subjects [8]–[21]. Due to technical limitations (i.e. number of recording amplifiers) and to the burden of post-hoc analysis, all the reports regard one (the vast majority) to a maximum of 4 upper limb individual muscles simultaneously analysed [10], [22], [23]; in this way a roughly “somatotopic gradient” similar to *penfieldian* organization -in which wide overlapping was observed- was consistently reported.

Different studies demonstrated both in primates and in humans that several loci along the pre-central motor strip contain separate representations of the same muscle (convergence). Meanwhile, it was shown that the same cortical site when focally stimulated can dispatch outgoing impulses to different muscles (divergence) and that muscles acting at different upper limb joints are represented at different cortical foci where they act in concert with different companion muscles. It has been shown that such parameters are highly influenced by limb posture [24]–[28]. Convergence, divergence, multiple muscle cortical representations and the influence of posture seem to play a pivotal role also in humans, orchestrating several muscles ‘in concert’ during motor actions, motor learning and post-lesional ‘plastic’ brain reorganization [29]–[32]. Even if invasive stimulation/recording procedures allowing a direct evaluation of these phenomena cannot be used in humans, TMS offers a valuable probe for a better knowledge of the dynamics of the cortical topography of individual muscles when they are treated as an *ensemble* and not as individual actors.

The aim of the present study was to investigate overlap and covariation of cortical motor output during TMS mapping, and simultaneous recordings from 12 individual upper limb muscles in order to measure cortical overlapping and covariation of pairs of muscles, interhemispheric differences and influence of arm/hand posture. Two measures were chosen to indicate overlap and covariation: 1) cortical overlapping of each pair of muscles, defined as the percentage of grid positions where TMS elicited a MEP response in both muscles respect to the total number of grid positions where TMS elicited a MEP response in at least one of the two, and 2) covariation of each pair of muscles, measured by means of Pearson's correlation. These measures make it possible to disentangle two neurophysiological aspects of functional connectivity: first, overlapping is related to the co-activation of the target muscles and indicates how much pairs of muscles are simultaneously represented in the motor cortex; second the covariation could be an index of the intensity and the direction of the co-activation.

## Results

**Figure 1:**
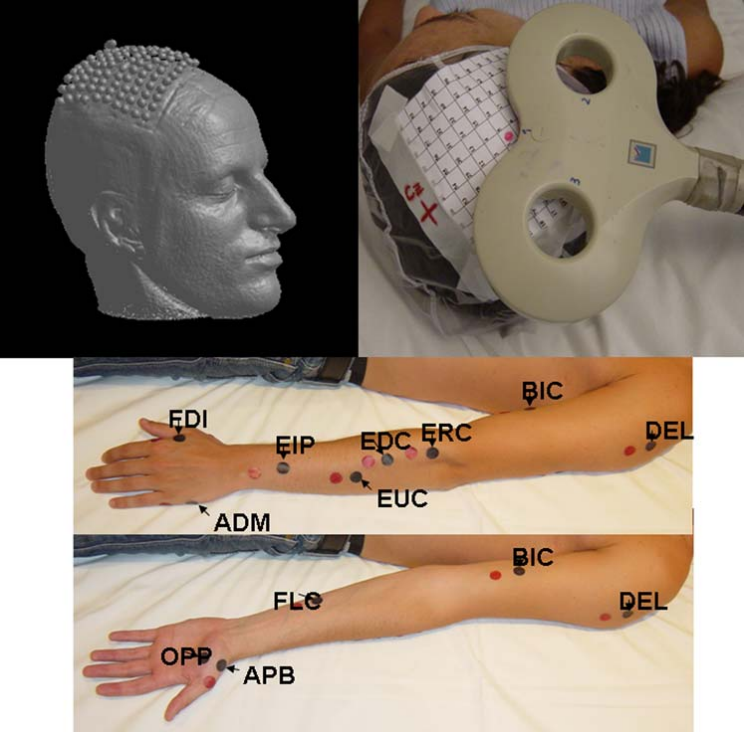
Experimental setting and electrode positioning for simultaneous MEP recordings from the examined muscles.

**Figure 2:**
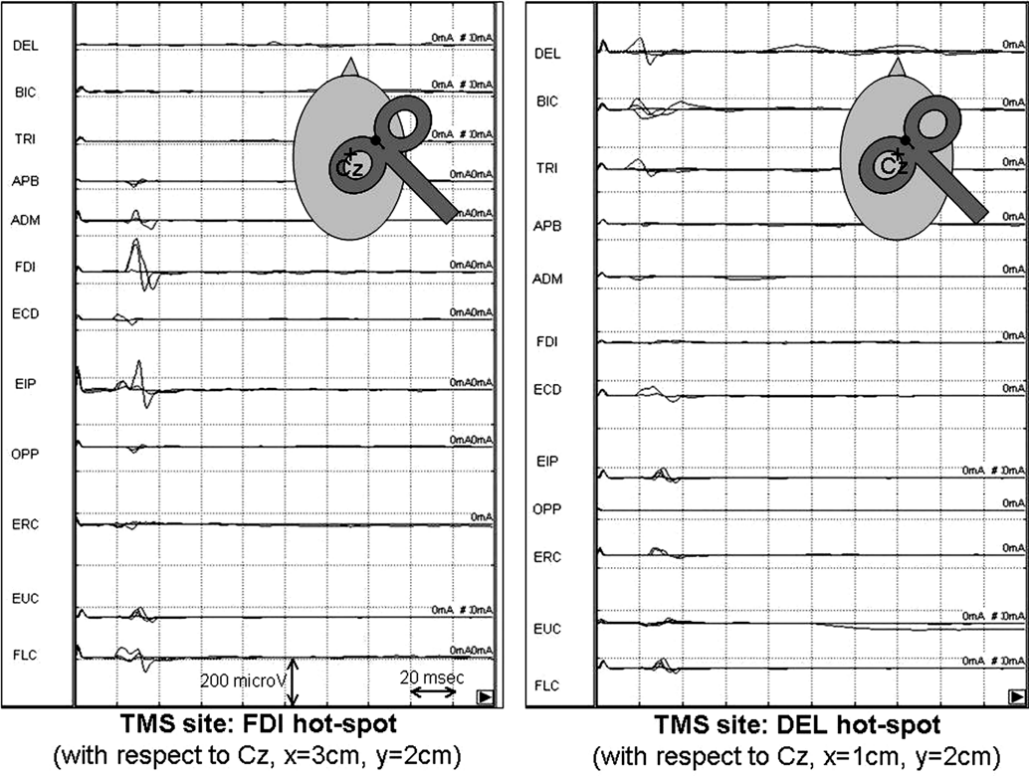
Original MEPs obtained from 12 muscles simultaneously at a two different stimulation sites, corresponding to: left panel, hot-spot of FDI (with respect to Cz, x = 3 cm, y = 2 cm); right panel, hot-spot of Deltoid (with respect to Cz, x = 1 cm, y = 2 cm); time and amplitude calibrations are 20 msec and 200 microV respectively.

The experimental setting and electrode positioning for simultaneous MEP recordings from the examined muscles is represented in Fig. 1, while traces of the original MEPs obtained from 12 muscles of subject 2 are shown in Fig. 2.

### Right hemisphere, left prone hand (10 subjects)

The 66 pairs of muscles showed significant differences for clusters of pairs sharing similar levels of cortical overlapping [F(65, 590) = 14.221, p<.001] as shown in Table 1 by Tukey's homogenous subsets (p value higher than 0.90).

**Table 1:** Percentage of overlapping between each of the 66 pairs of muscles.

	Homogeneous subsets (Tukey's procedure)						
*Pairs*	1	2	3	4	5	6	7
*EIP-TRI*	12						
*DEL-EIP*	12						
*DEL-FDI*	15						
*DEL-OPP*	15						
*DEL-EUC*	15						
*BIC-EIP*	15						
*ADM-DEL*	16						
*ERC-TRI*	16						
*DEL-ERC*	16						
*DEL-EDC*	16						
*DEL-FLC*	17						
*FDI-TRI*	17						
*ABD-DEL*	17						
*OPP-TRI*	17						
*EUC-TRI*	17						
*ADM-TRI*	18						
*FLC-TRI*	18						
*DEL-TRI*	18						
*EDC-TRI*	19						
*ABD-TRI*	19						
*BIC-FDI*	20						
*BIC-OPP*	21						
*BIC-ERC*	21						
*BIC-EUC*	21						
*ADM-BIC*	22						
*BIC-EDC*	22						
*ABD-BIC*	23						
*BIC-DEL*	23						
*BIC-FLC*	24	24					
*BIC-TRI*	29	29	29				
*EIP-ERC*		56	56	56			
*EDC-EIP*			58	58	58		
*EIP-FLC*				62	62	62	
*EIP-OPP*				64	64	64	
*EIP-EUC*				64	64	64	64
*ADM-EIP*				67	67	67	67
*ABD-EIP*				67	67	67	67
*ERC-FDI*				69	69	69	69
*EDC-ERC*				72	72	72	72
*EIP-FDI*				72	72	72	72
*ERC-FLC*				73	73	73	73
*EDC-FDI*				74	74	74	74
*ERC-OPP*				74	74	74	74
*ERC-EUC*				75	75	75	75
*ADM-ERC*				76	76	76	76
*FDI-FLC*				77	77	77	77
*ABD-ERC*				78	78	78	78
*EDC-FLC*				80	80	80	80
*ABD-FDI*				82	82	82	82
*EDC-EUC*				82	82	82	82
*EDC-OPP*				83	83	83	83
*ADM-EDC*				84	84	84	84
*FLC-OPP*				84	84	84	84
*EUC-FLC*				85	85	85	85
*EUC-FDI*				85	85	85	85
*ABD-EDC*				86	86	86	86
*ADM-FLC*				86	86	86	86
*FDI-OPP*				86	86	86	86
*ADM-FDI*				86	86	86	86
*ABD-FLC*				87	87	87	87
*ABD-EUC*					90	90	90
*ABD-OPP*					90	90	90
*ABD-ADM*						91	91
*ADM-EUC*						93	93
*ADM-OPP*						94	94
*EUC-OPP*							96
***p-value***	***0.999***	***0.906***	***0.978***	***0.951***	***0.922***	***0.931***	***0.901***

**Figure 3:**
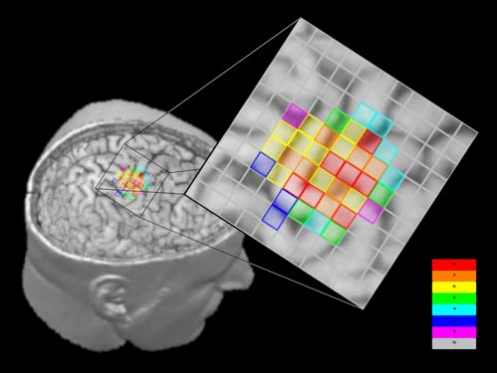
TMS-MRI integration: number of co-activated muscles for each scalp position in a paradigmatic subject.

A first subset is characterised by a low overlapping level (range: 12% for EIP-TRI, 29% for TRI-BIC) and is clearly identified by all pairs including at least one of the three arm muscles. A second group of pairs is characterized by a high overlapping level (range: 64% for EIP-EUC, 96% for EUC-OPP) which comprises almost all hand-hand, hand-forearm and forearm-forearm pairs. Between these two subsets, there is not a unique and well defined subset, but it can be noted that all pairs are characterised by the presence of EIP muscle: the cortical representation of this muscle overlaps with the other forearm and hand muscles in a range between 56% and 67%, reaching the maximum with FDI (72%). The number of co-activated muscles for each scalp position in one paradigmatic subject is represented in Fig. 3.

**Figure 4:**
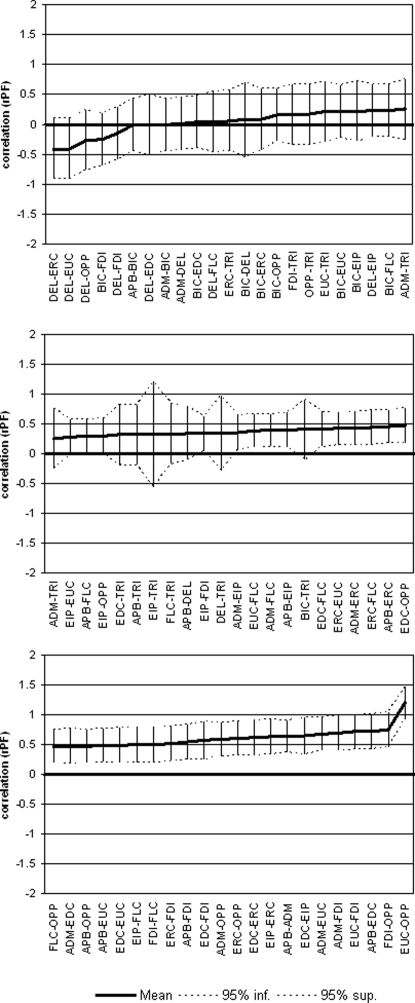
Covariation measure of each pair between the 12 examined muscles. This measure was obtained from the right hemisphere of 10 healthy subjects lying with prone hands.

Similar analysis was applied to the other measure of association. In this case, we also tested the significance of the correlation index for each pair of muscles with respect to the lack of correlation (0 value). When the 95% confidence intervals did not include the 0 reference line, a correlation (negative or positive) can be documented (with α error at 5%). The size of the correlation index changed according to which specific pair of muscles was considered [ANOVA F(65, 367) = 2.205, p<.001]. Looking at Tukey's homogenous subsets, two main subsets were identifiable: 1) pairs including at least one arm muscle; 2) pairs including hand and forearm muscles. However, the standard error of the mean for this index is much larger than for the overlapping one and it was more difficult to assess whether some pairs were statistically more correlated than others. Meanwhile, graphical representation with 95% error bars (Fig. 4) showed that in the first subset (pairs including at least one arm muscle) correlation always crossed the 0 reference line (lack of correlation), while the second subset was consistently characterised by high correlation levels up to the most correlated pair, i.e EUC-OPP (untransformed r = 0.786).

The patterns observed for the two measures of cortical association in the right hemisphere were used as baseline *vs* the left hemisphere and when the influence of the hand position (prone *vs* supine) was analysed.

### Comparison between right and left hemispheres (right prone hand: 6 subjects)

**Figure 5:**
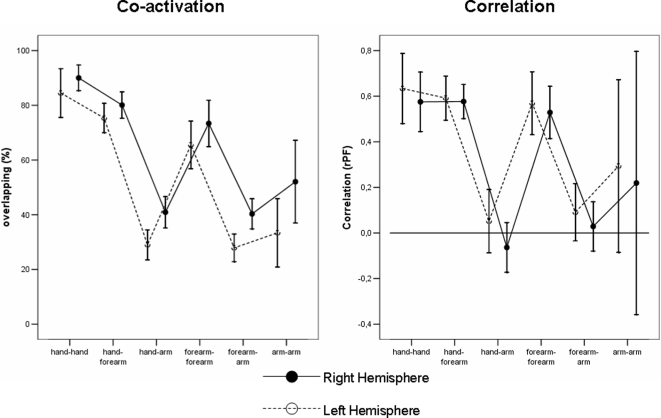
Inter-hemispheric comparison of co-activation and covariation measures (obtained with hand in prone position) between pairs of muscles belonging to three different districts (hand, forearm, arm).

When right and left hemispheres were compared in terms of co-activation –even if with a quite similar pattern of muscle associations (Fig. 5, right panel)- a significant *Hemisphere* effect was found [F(1, 659) = 21.199, p<.001], indicating that the overlapping between all possible pairs of muscles was significantly higher in the left (mean overlapping 62.5%; 95% CI = 59.8–65.3) than in the right hemisphere (mean overlapping 53.5; 95% CI = 50.8–56.2) (Fig. 5). No interaction *Hemisphere*×*Muscle_pairs* was observed [F(65, 659) = 0.536, p = .999] also when pairs were grouped according to the upper limb district [F(5,779) = 0.817, p = .538].

A different pattern was observed when the covariation measure was analysed. In this case, no evidence of higher association in left *vs* right hemisphere was found [F(1,526) = 1.515, p = .219], mean correlation being 0.37 (95% CI = 0.31–0.43) in the latter and 0.32 (95% CI: 0.27–0.37) in the former. Since higher correlation -although not significant- was observed in the right hemisphere, further analysis was performed in order to compare the two association measures in the two hemispheres (after converting them into a common scale, i.e. z-scores). This analysis indicated a significant *Association_measure*×*Hemisphere* interaction [F(1,656) = 7.014; p<.008], supporting the hypothesis of a different kind of association between muscles in the two hemispheres.

### Comparison between supine and prone hand (left hand-right hemisphere,3 subjects)

**Figure 6:**
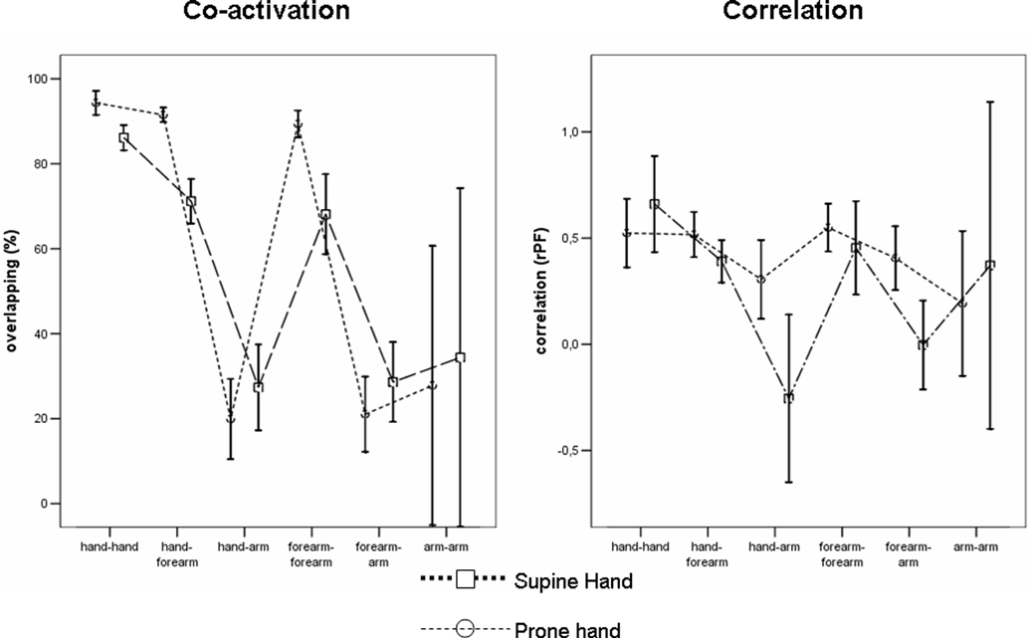
Inter-position comparison of co-activation and covariation measures (obtained from the right hemisphere) between pairs of muscles belonging to three different districts (hand, forearm, arm).

Hand posture influenced both measures of cortical association. In fact, the effect of *Position* was significant [F(1,392) = 5.419, p = .021] on co-activation with a 59.5% overlap (95% CI = 55.8–63.3) with prone hand and a 53.2% overlap (95% CI = 49.4–57.0) with supine hand, respectively. However, the larger overlapping with prone position was specifically evident for hand and forearm muscle pairs, while no changes occurred in the overlapping of proximal muscles with the more distal ones (Fig. 6).

The correlation between pairs of muscles resulted higher with prone than with supine hand [F(1,160) = 7.025, p = .009]. The presence of a significant *Couple_district*×*Position* interaction [F(5,280) = 2.739, p = .020] could be ascribed to the lower correlation between proximal and distal districts in case of supine hand.

## Discussion

Modern concepts on the functional organization of cortical primary motor areas maintain a somatotopic view only for major body districts (such as face, arm, leg), each being sustained by a network serving broadly distributed functions which involve large populations of neurons in spatially separate clusters. Within this theoretical frame, the main goal of the present study was to investigate whether the use of a non-invasive technique like TMS could roughly disentangle the representational overlapping between different upper limb muscles and the eventual functional correlation in humans. [33]–[37].

The existence of multiple yet discrete efferent micro- and macrozones from primary motor cortex, is now accepted as the essential organizational principle of this area. Animal studies have in fact demonstrated that a particular movement can be elicited through stimulation of different M1 regions, often several millimeters apart and separated by non-responsive districts [24]–[27], [39]–[43]. In addition, bi-directional projections interconnect motor cortex areas for different muscle districts [24], [44] for a review. Any movement therefore, differently from the traditional view of the *labelled-line* hypothesis, is probably not controlled by single aggregates, but by a distributed network within the M1 cortex. In this model, the motor output from overlapping cortical territories converge onto individual muscles. Similarly, the output from any given cortical site diverges onto multiple muscles with different ‘gain’ according to the final movement to be performed, presumably regulated by horizontal intracortical projections interconnecting functionally related neuronal clusters within M1 [43], [45], [46].

Multiple representations of the cortical output to individual muscles, overlap each other. Multiple cortical representation of muscles controlling hand/fingers, represents the functional frame for the extraordinary repertoire of movement strategies (i.e. concerted actions orchestrated by many –sometimes the same- muscles acting on different joints at different times [24], [37], [47]). Such an organization also provides flexibility in motor planning and execution, and progressively substitutes a related dysfunctional area more easily than highly specialized and unique groups of cells, being thus fundamental either for restorative or for maladaptive plasticity.

Precise methods such as those in experimental models, allowing microneurographic stimulation/recording procedures from individual neurons/fibers in the motor cortex, pyramidal tract and spinal motoneurons cannot be used in humans to address physiological mechanisms for movement control like convergence, divergence and multiple muscle representation. Several techniques for functional brain imaging –namely PET and fMRI- allow detailed images on the relationship between function and anatomy showing the distributed network subtending a given motor act. However they cannot discriminate the temporal dynamics of a phenomenon taking place in few tens of milliseconds or differentiate neuronal firing decrease from increase (exciting *vs* inhibiting net effects); moreover, in the case of movement paradigms, the activation directly linked to motor programming and execution cannot be distinguished from the sensory input feed-back from the moving parts, and the chronological hierarchy –if any- governing activated areas cannot be studied appropriately [48], [49]. Despite their potential ability to properly follow-up such a brain mechanism, non-invasive brain stimulation procedures have never been tested along this vein in human beings, despite their progressively growing use in research for movement physiology and pathophysiology see [7] for a review. In fact, transcranial magnetic stimulation of the primary motor cortex has largely been employed in investigating neurophysiological mechanisms of motor control in the healthy subjects[9], [11], [18], [19], [50]–[65] and in patients with neurological motor deficits [13], [15], [66]–[71]. Mapping procedures of motor cortical output have been progressively implemented and integrated with precise anatomical brain reconstructions [23], [32], [33], [72]–[78]. However, no previous studies investigated in detail the functional linkage amongst upper limb muscles simultaneously recorded during TMS mapping of output from M1 cortex.

We document significant overlapping between proximal and distal muscles, in line with many animal studies involving the superposition of topographical maps of the motor cortex obtained by microstimulation and morphological connectivity maps obtained by tracer injections. The concordance between such studies and our TMS findings (related to trans-synaptic activation) could also be referred to the existence of intrinsic horizontal collaterals (intracortical connections) [79].

Findings from the present study demonstrate significant difference between the 66 pairs of muscles in terms of cortical overlapping: this index reached high scores for hand-forearm muscles and low for arm *vs* hand/forearm muscles. Moreover, a subset of low-overlapping muscles characterised by a low percentage of overlap was clearly identified by all pairs with at least one of the three arm muscles. In a separate cluster, there is a group of pairs with high-overlapping muscles which include almost all hand-hand, hand-forearm and forearm-forearm possible pairs.

When covariation was taken into consideration interesting observations emerged. In particular, our data were obtained at rest and there was a lack of covariation between forearm/hand and more proximal arm muscles. Our covariation measure gives an evidence of the motor cortex representation of “muscle synergies” (in terms of the directionality of a coactivation) that should not be considered hard-wired entities. In fact, in the modern view of an integrated nature of motor cortical function, movement-related muscle synergies “may be dynamically created by the operations of motor cortical circuitry” [79]–[80].

Moreover, high covariations for wrist extensors and hand muscles were seen up to the most correlated pair EUC-OPP. One might argue that this functional organization of M1 output is strongly devoted to the control of wrist joint.

When right and left hemispheres were compared, a significant *Hemisphere* effect was found indicating that the index of overlapping in the left was significantly higher than in the right hemisphere. Even if this observation is intriguing, whether it has some relationship with manual dexterity and hemispheric dominance for hand motor preference, needs further evaluation in a population with right *vs* left-hand dexterity. It is worth noting that a larger overlapping in the left hemisphere, without a corresponding inter-hemispheric difference of the single muscle cortical representation was found. This larger overlapping in the left hemisphere could be related to its lifetime higher training of all twelve studied muscles with respect to the right hemisphere, resulting in a larger connectivity within M1 cortex.

Interhemispheric difference was not observed when the covariation measure was analysed. Covariation could be interpreted as an index of the intensity and direction of the co-activation; therefore, the larger overlapping observed in the left hemisphere did not involve a higher co-variation.

Of course, covariation/overlapping measures are significantly correlated (r = 0.49), indicating that about 25% of the variance of one could be accounted for by the other. In fact, to calculate the covariation the two muscles have to be simultaneously represented in the motor cortex (that is to say, there must be coactivation/overlapping of the two muscles). So we can affirm that overlapping is the basic phenomenon on which the covariation takes place, but also that covariation could be considered as an index of the intensity and direction of the co-activation. In other words, co-activation and covariation could be summarised only partially by one of them. The contamination between these two measures, although present, is not sufficient to conclude that they indicated the same phenomenon. In addition, the similarity of patterns represented in the two panels of Fig. 5 is only apparent, since co-activation was higher in right than in left hemisphere (left panel), while covariation was not different in the two hemispheres. For these reasons (the first neurophysiological, the second statistical) the two measures can reflect two different neurophysiological aspects of functional connectivity.

Network structure can adjust dynamically to meet the immediate needs of the motor system, as seen in response to postural changes [36]; indeed, in the present study hand position significantly influenced both overlapping and covariation indexes. The larger overlapping with prone hand seemed to be specific to pairs of muscles belonging to hand and forearm, while no changes occurred for proximal muscles. Therefore, this effect seemed specific to the muscles and districts where the main change in proprioceptive input occurred, and can be linked to task-devoted organization of synaptic contacts at the periphery of cortical maps of the muscles involved. Altogether, present findings with prone hand might be ascribed to cortical mechanisms facilitating coupling of muscle pairs for object' grasping and lifting -which needs involvement of more proximal muscles for joint stabilization and increased upper limb stiffness- compared to supine hand, facilitating a motor action most apt for object catching on a vertical axis, with less proximal muscle involvement [81].

The potential effect of different peripheral nerve territories involvement on association measures was also examined, but neither overlapping nor covariation indexes were statistically linked to muscle pairs innervated by branches belonging to the same nerve trunk respect to the pairs controlled by different nerves. This indicated that the observed patterns of muscle associations, take place within CNS, even if the present approach cannot discriminate between cortical and subcortical (including spinal) contributions.

In conclusion, simultaneous upper limb multiple-muscle recordings during mapping of M1 output with focal TMS has provided a bulk of information strongly related to the functionality of the arm/hand system in healthy humans. This approach will hopefully permit better understanding of upper limb motor control in healthy subjects and in patients with motor disorders, by providing prognostic indexes for motor recovery and in following-up ‘plastic’ reorganization of the motor system after acute or progressive lesions.

## Materials and Methods

Mapping studies with TMS were performed on 10 healthy volunteers (6 females and 4 males), right handed, aged 28–40. The experimental protocol had the approval of the ethics committee of Fatebenefratelli Hospital-Isola Tiberina (Rome) and all subjects gave written informed consent; international safety standards –including an EEG before subject were recruited for TMS- were strictly followed [20], [82].

Simultaneous EMG records were obtained from the following 12 muscles in different experimental conditions: *Abductor Digiti Minimi* (ADM), *First Interosseus Dorsalis* (FDI), *Opponens Pollicis* (OPP), *Abductor Pollicis Brevis* (APB), *Extensor Digitorum Communis* (EDC), *Extensor Indicis Proprius* (EIP), *Extensor Carpi Ulnaris* (EUC), *Extensor Carpi Radialis* (ERC), *Flexor digitorum superficialis (communis)* (FLC), *Biceps brachii* (BIC), *Triceps* (TRI), *Deltoid* (DEL).

During the experimental session the subject, wearing a transparent and well-fitting plastic cap, lay supine on a bed in order to facilitate complete relaxation and was kept alert by the investigator.

Six subjects attended for two testing procedures, one for each hemisphere, with arm, forearm and hand relaxed in prone position. In three of them, another recording session was carried out only for right hemisphere with arm, forearm and hand relaxed in supine position. This further recording was performed in order to assess eventual modifications induced on cortical somatotopy by arm/hand posture. The remaining subjects only had one hemisphere and one hand position studied.

Focal single-pulse TMS was delivered to the scalp using a flat figure-of-eight coil with an inner diameter of 70 mm for each wing connected to a Magstim 200 magnetic stimulator (Magstim Company, Whitland, UK).

The coil was placed tangentially to the skull with the handle pointing backward and rotated away from the midline by 45°.

Stimulus intensity was adjusted at resting motor threshold defined on the hot spot following international guidelines as the stimulator's output able to elicit reproducible MEPs (at least 50 µV in amplitude) in about 50% of 10–20 consecutive stimuli in a completely relaxed Opponens Pollicis muscle [20], [82]. Once the excitability threshold was determined, TMS intensity was increased by 10% of the stimulator output to enhance response probability. This intensity was an obvious compromise between the different excitability thresholds of the upper limb muscles and is usually able to elicit responses with a decreasing probability from hand to shoulder muscles (the latter having a higher threshold and stronger intensities requirement). This was a forced choice in order to avoid an excessive “cortical spread” of the stimulus which would have occurred if we had utilized 110% intensity of the threshold of the less excitable (i.e. arm/shoulder) muscles.

The hot spot of the OPP, defined as the point from which stimuli triggered Motor Evoked Potentials (MEPs) of maximal amplitude and minimal latency, was found during a pilot stimulating/recording session with a frankly suprathreshold stimulus intensity. The hot spot position was then marked on a transparent, adherent elastic cap to facilitate an exact re-positioning of the coil during the entire experiment. In one paradigmatic subject 3D head/brain Magnetic Resonance Imaging (MRI) was obtained, coregistered with the grid of recording scalp electrodes as represented by vitamin E capsules. Therefore, data presented in Fig. 3 are realistic and not merely a graphical representation.

Using surface electrodes, EMG activity was recorded simultaneously from each of the 12 target muscles. Skin was cleaned at the recording site followed by placement of Ag/AgCl cup electrodes filled with conductive gel in a belly/tendon montage. Recording electrodes were placed over the bellies (active) and tendons (reference) of muscles to be monitored and ground a electrode was placed over the wrist (Fig. 1). Interelectrode distance was maintained as short as possible; this -together with the TMS intensity just above threshold- was deliberately chosen in order to reduce the amount of cross-talk from muscles different and adjacent from the one under examination. Skin/electrode resistances were kept <10 KOhms.

Signal recording was carried out with a modified version of the BASIS-Brain Surveyor Equipment (EB-Neuro; 12 channels; sampling rate 5 KHz/channel, filtering bandpass 3–1500 Hz).

Subjects were wearing an elastic, transparent cap containing a grid of 121 numbered squares marked at 1 cm intervals, in which square 1 corresponded to the designated hot-spot of the OPP hand muscle used to define the excitability threshold. The remaining squares were subsequently numbered in a spiral fashion. The scalp vertex (the point on the midsagittal line, midway between nasion and inion) and the scalp anatomical landmarks (nasion, inion) were taped in a stable position on the cap.

Four consecutive MEPs were gathered from each grid position starting from square 1 and following the progressive numbering with a stimulus repetition rate of 0.1–0.2 c/sec. The progression of stimulated sites of the scalp grid followed a spiral sequence, that could be considered a relatively safe method to avoid sequential stimulation of an ordered and topographically organized cortical representation of the muscles.

MEPs latency and amplitude were measured at peak onset and peak-to-peak of opposite polarity, respectively; they were calculated off-line for each stimulated site and for each muscle. Particular care was devoted to maintain full relaxation of the examined muscles and trials contaminated by involuntary movement or muscle activity were discarded from further analysis (Fig. 2).

### Data analysis

A data matrix containing all raw latencies and amplitudes of MEPs was prepared for each of the 12 muscles elicited by stimulation on each of 121 scalp positions corresponding to the 11×11 grid, centred around the hot-spot. Since for each position, four successive stimuli were delivered, the matrix contained 424 rows for each subject. Ten subjects underwent TMS of the right hemisphere (with prone left hand), 6 subjects underwent the TMS of both hemispheres (with prone hands) and 3 subjects underwent the TMS of right hemisphere (with prone and supine left hand). Altogether 20 different TMS sessions entered data analysis. Therefore, the complete raw dataset consisted of 8056 rows and 27 columns (the first three to identify subjects, hemisphere and hand position, then 12 for latencies and 12 for amplitudes). It is worth noticing that a number of the matrix cells were empty, since no MEP was obtained from the corresponding scalp positions and muscles.

In order to reduce the large variability of MEP amplitude data, *log* transformation was applied [83]. In addition, the median value was considered for each scalp position: since four stimuli were delivered on each stimulation site, median value was the arithmetic mean in the case two MEPs were obtained, the unique central value in the case three MEPs were obtained, the arithmetic mean of the two central values after discarding the two extremes in case four MEPs were obtained. When just one MEP was observable, the amplitude was set at a missing value.

### Measures for cortical associations between muscle representations

Many studies faced the issue of mapping the motor area and nowadays each mapping study should rely on a neuronavigation tool allowing the integration between TMS and neuroimaging techniques. Thanks to such procedures, the cortical motor area of each muscle could be rigorously described and analysed by means of parameters characterizing such areas as excitability (number and extension of “active” points on the scalp), volume (sum of MEP amplitudes obtained stimulating the whole active area), center-of-gravity (the amplitude-weighted mean position) [10], [14], [76], [84]. These parameters make it possible to address the issues of “convergence” (TMS on several different scalp positions produce a MEP in the same target muscle), “divergence” (TMS on a single scalp position produce a MEP in different target muscles) and somatotopy of motor area (based mainly on hot-spot and centre-of-gravity positions). It is however worth remembering that the aim of the present study is not to replicate previous mapping findings, but to describe and investigate the association between upper limb muscles in terms of their cortical representation. Since multivariate techniques, such as Multi-Dimensional Scaling (MDS) did not provide consistent findings, two simple measures of association between muscles were computed:

1. cortical overlapping of each pair of muscles, defined as the percentage of grid positions where TMS elicited a MEP response in both muscles respect to the total number of grid positions where TMS elicited a MEP response in at least one of the two muscles. For example, the overlapping between FDI and ADM was: N_FDI∩ADM_/N_FDI∪ADM_.
2. cortical covariation of each pair of muscles, measured by means of Pearson's correlation based on MEP amplitude. To be noted that Pearson's correlation were transformed according to Fisher's transformation 0.5*ln((1−r)/(1+r)) in order to obtain a measure more suitable for statistical tests (that will be denoted hereafter *rPF*).

Neurophysiological interpretation of cortical overlapping mainly relies on co-activation of the target muscles and indicates how much two muscles are simultaneously represented in the motor cortex. On the other hand, covariation could be interpreted as an index of the intensity and direction of co-activation: for example, a negative correlation between two muscles would result when high MEP amplitude in one muscle corresponded to low MEP amplitude in the other and vice versa. In other words, this would be the case when, although simultaneously represented, a MEP in one muscle of a pair is elicited by recruitment of few 1^st^ and 2^nd^ motoneurons (small response with respect to the average) while the other is elicited by a larger pool of motoneurons (large response with respect to the average), provided the same scalp site is stimulated in both conditions.

Since 12 muscles were triggered by each stimulus, 66 pairs could be defined. During the statistical analysis and for graphic presentation, muscles were grouped in three districts (4 hand muscles-Group I, 5 forearm muscles-Group II, 3 arm muscles-Group III). The 66 muscle pairs were therefore distributed as follows: 6 hand-hand, 20 hand-forearm, 12 hand-arm, 10 forearm-forearm, 15 forearm-arm, 3 arm-arm.
